# Soil CO_2_ emissions in cropland with fodder maize (*Zea mays* L.) with and without riparian buffer strips of differing vegetation

**DOI:** 10.1007/s10457-022-00756-5

**Published:** 2022-07-22

**Authors:** J. C. Dlamini, L. M. Cardenas, E. H. Tesfamariam, R. M. Dunn, J. Evans, J. M. B. Hawkins, M. S. A. Blackwell, A. L. Collins

**Affiliations:** 1 Department of Soil, Crop and Climate Sciences, University of the Free State, Bloemfontein, 9300 South Africa; 2 Sustainable Agriculture Sciences, Rothamsted Research, North Wyke, Okehampton, Devon, EX20 2SB UK; 3 Department of Plant and Soil Sciences, University of Pretoria, Private Bag X20, Hatfield, 0028 South Africa; 4 Computational and Analytical Sciences, Rothamsted Research, West Common, Harpenden, Hertfordshire, AL5 2JQ UK

**Keywords:** Freshwater courses, Arable land, Carbon dynamics, Mineralisation

## Abstract

Vegetated land areas play a significant role in determining the fate of carbon (C) in the global C cycle. Riparian buffer vegetation is primarily implemented for water quality purposes as they attenuate pollutants from immediately adjacent croplands before reaching freashwater systems. However, their prevailing conditions may sometimes promote the production and subsequent emissions of soil carbon dioxide (CO_2_). Despite this, the understanding of soil CO_2_ emissions from riparian buffer vegetation and a direct comparison with adjacent croplands they serve remain elusive. In order to quantify the extent of CO_2_ emissions in such an agro system, we measured CO_2_ emissions simultaneously with soil and environmental variables for six months in a replicated plot-scale facility comprising of maize cropping served by three vegetated riparian buffers, namely: (i) a novel grass riparian buffer; (ii) a willow riparian buffer, and; (iii) a woodland riparian buffer. These buffered treatments were compared with a no-buffer control. The woodland (322.9 ± 3.1 kg ha^− 1^) and grass (285 ± 2.7 kg ha^− 1^) riparian buffer treatments (not significant to each other) generated significantly (*p = < 0.0001*) the largest CO_2_ compared to the remainder of the treatments. Our results suggest that during maize production in general, the woodland and grass riparian buffers serving a maize crop pose a CO_2_ threat. The results of the current study point to the need to consider the benefits for gaseous emissions of mitigation measures conventionally implemented for improving the sustainability of water resources.

## Introduction

Vegetated land areas play a pivotal role in understanding carbon (C) dynamics in the global C cycle (Stauch et al. 2008). Vegetated riparian buffer strips are primarily introduced between croplands and waterbodies to attenuate non-point source (NPS) pollutants from agricultural lands from reaching freshwater ecosystems (Jaynes and Isenhart 2014; Lowrance et al. 2002; Valkama et al. 2018). The vegetated riparian buffers usually recycle high organic matter that elevates soil C and are usually anoxic since they sustain high soil moisture from seasonally high water tables (Jacinthe 2015). These conditions, as mentioned above, and the processing of the pollutants promote biological processes including denitrification, mineralization, and fermentation, which produce greenhouse gases, including carbon dioxide (CO_2_) (Kayranli et al. 2010; Thangarajan et al. 2013).

Soil CO_2_ production and subsequent emissions indicate soil respiration in the biota, as both are influenced by factors controlling CO_2_ movement in the soil (Raich and Potter 1995; Raich and Schlesinger 1992). Soil temperature and moisture are considered the most dominant factors influencing soil CO_2_, as they influence CO_2_-producing soil biological activities (Davidson et al. 1998). Soil organic matter provides a substrate for soil CO_2_ producing microbial activities, and its decomposition result in CO_2_ production in soils (Harrison-Kirk et al. 2013); thus, it is expected that vegetation that recycles the most organic matter might have high CO_2_ production. However, this may be highly dependent on the labile C fraction, as Dlamini et al. (2020) observed that soils containing highly labile C result in high CO_2_.

Previous studies, De Carlo et al. (2019) and Jacinthe (2015), have compared CO_2_ emissions from different riparian buffer vegetation types. Despite previous work, understanding of CO_2_ fluxes and their controlling soil and environmental variables from riparian buffer strips and a direct comparison with a cropland they serve remain elusive. Therefore, this study aimed to evaluate the unintended emissions of CO_2_ through through the soil respiration process and enrich the understanding of their soil and environmental controls from maize production, which had both buffered and un-buffered downslope.

## Materials and methods

### Experimental site

The replicated plots used in this experiment are located at Rothamsted Research, North Wyke, Devon, United Kingdom (50°46 × 10″ N, 3° 54 × 05″ E). The area is situated at an altitude of 177 m above sea level, has a 37-year (from 1982 to 2018) mean annual precipitation (MAP) of 1033 mm (with the majority of rainfall received between October and November of each year), and mean annual temperature (MAT) of 10.1 °C (Orr et al. 2016). The experimental area has a slope of 8° and is on soils of the Hallsworth series (Clayden and Hollis 1985), or a dystric gleysol (FAO 2006), with a stony clay loam topsoil comprising of 15.7% sand, 47.7% clay, and 36.6% silt (Armstrong and Garwood 1991) overlying a mottled stony clay, derived from Carboniferous Culm rocks. The subsoil is impermeable to water and is seasonally waterlogged; most excess water moves by surface and sub-surface lateral flow across the clay layer (Orr et al. 2016), thereby making replicated experimental work using hydrologically-isolated plots feasible.

### Experimental design and treatments

#### Experimental set-up

The experiment was laid out as three blocks of four plots corresponding to four treatments each. Each plot consisted of the main maize crop area with one gas measurement chamber and either a control (no-buffer) with a single chamber or a buffer area (sown with one of three different vegetation types) that had two chambers (upper and lower). The three buffered treatments comprised grass, willow, and woodland. Each of the four treatments was replicated three times, making a total of twelve plots. Each plot was 46 m in length and 10 m wide; the main upslope maize cropped area being 34 m in length (340 m^2^) and the downslope buffer strip being 12 m (120 m^2^) (see description below). To hydrologically-isolate each plot, a plastic-lined and gravel-filled trench was installed to a depth of 1.40 m to avoid the lateral flow of water and associated pollutants. The cropped upslope area was previously managed as a silage crop, with a permanent pasture dominated by ryegrass (*Lolium perenne* L.), Yorkshire fog (*Holcus lanatus* L.) and creeping bentgrass (*Agrostis stolonifera* L.) planted in 2016 which was ripped and ploughed on the 14th of May 2019 in preparation to plant maize whilst the riparian buffer areas remained untouched. Maize (*Zea mays* L.) was planted on the 17th of May 2019 for the experiment reported herein. Cattle slurry and inorganic fertilizer were applied at times and rates summarised in Table 1.

**Table 1:** Application rates of cattle slurry and inorganic fertilizer during the cropping season

Date	Application	N-input (kg ha^− 1^)	P-input (kg ha^− 1^)	K-input (kg ha^− 1^)
14 May 2019	Cattle slurry	20.8	12	46
17 May 2019	Inorganic fertilizer	100^a^	85^b^	205^c^

Nutrient sources: Nitrogen; ^a^Nitram (Ammonium nitrate), Phosphorus; ^b^ triple superphosphate (P_2_O_5_), Potassium^c^ muriate of potash (K_2_O)

#### Treatments description

(i) No-buffer strip control: plots without the 12 m x 10 m buffer strips. The area of land described as a no-buffer control was always managed precisely as the upslope maize crop.
(ii) Grass riparian buffer strip: Novel grass buffer strip (*Festulolium loliaceum* cv. Prior) - The novel grass was planted at the end of 2016 at a seeding rate of 5 kg ha^− 1^, a recommended seeding rate for the species in the Devon county. The novel grass hybrid was developed to be a dual-use grass species that provides efficient forage and could help mitigate flooding by increasing water infiltration (Macleod et al. 2013). During the current experiment, the 3-year old hybrid grass was about 80-cm tall and had never been cut since planting in 2016.
(iii) Woodland riparian buffer strip: Deciduous woodland - Six species, namely pedunculate oak (*Quercus robur* L.), hazel (*Corylus avellana* L.), hornbeam (*Carpinus betulus* L.), small-leaved lime (*Tilia cordata* Mill.), sweet chestnut (*Castanea sativa* Mill.), and wych elm (*Ulmus glabra* Huds.) were planted in the woodland buffer strips. Five individual plants of each species were bare-root planted at the end of 2016 within the 10 × 10 m buffer strip area, plant density of 3000 plants ha^− 1^; a recommended planting density for the Devon county. The woodland species were chosen for their ability to respond well to coppicing (whereby the wood is cut to near ground level and the tree sends out new shoots to form a stool the next growing season). The choice was also based on financial incentives for planting woodland along buffer zones and, as well as it’s potential for water quality improvement (Sydes and Grime 1981). This choice also fitted with the local agri-environment payment scheme available at the time (Countryside Stewardship) for a riparian buffer zone, so it would be something that farmers with watercourses would be able to receive a payment for, in terms of getting money to plant the trees in their riparian areas. During the current experiment, the 3-year old woodland trees were 1.6 m tall and had never been cut since planting in 2016.
(iv) Willow riparian buffer strip: Bio-energy crop - five willow cultivars namely Cheviot, Mourne, Hambleton, Endurance and Terra Nova (all *Salix* spp.); the first three being newly developed cultivars and the latter being older ones. These were planted as 30 cm long whips in May 2016 at a population of 200 plants per 10 m x 10 m area, plant density of 20,000 plants ha^− 1^; a recommended planting density for willows in the Devon area. The willow cultivars were chosen from the National Willow Collection based at Rothamsted Research, Harpenden site to be suitable for growing in the wet clay-rich soils of the Devon site. They were also chosen based on their high capacity for pollutant uptake and their use for soil bioremediation (Aronsson and Perttu 2001). During soil sampling for the current incubation experiment, the 3-year old willow trees were about 3-m tall and had not been cut since planting in 2016.

Each of the three riparian buffer strip areas were sprayed with glyphosate herbicide to remove pre-existing grassland vegetation to enable better establishment of the planted deep rooting grass (*Festulolium loliaceum* cv. Prior), willow and woodland trees. The deep rooting grass buffer strips were also rotavated prior to seed broadcast. Each of the buffer strips was comprised of two parts – the lower slope area comprised a 2-m strip of natural grass, with the upslope area comprising a 10-m strip of treated and planted vegetation. The lower slope area of 2-m natural grass strip is the requirement for cross-compliance in England whereby farmers with watercourses must adhere to Good Agricultural and Environmental Condition (GAEC) rule 1; establishment of buffer strips along watercourses (DEFRA 2019). The 10 m x 10 m area (10-m width) is the GAEC recommended N fertilizer application limit away from surface waters.

### Field measurements and laboratory analyses

#### CO_2_ measurements

##### Field sampling and analyses

Carbon dioxide fluxes were measured using the static chamber technique (Chadwick et al. 2014; De Klein and Harvey 2012). The polyvinyl chloride (PVC) chambers were square frames with lids (40 cm width x 40 cm length x 25 cm height) with an internal base area of 0.16 m^2^. Thirty-three chamber collars were inserted to a depth of 5 cm below the soil surface using a steel base, and installation points were marked using a hand-held global positioning system (GPS; Trimble, California, USA) so that they could be moved into the same positions after periodic removal for agronomic activities (e.g., tillage). In the willow and woodland riparian buffers, maize cropped areas, and no-buffer control, chambers were installed in-between two rows, while in the grass riparian buffers, chambers were installed in pre-determined positions. More specifically, the chambers were positioned as follows: (i) in area ‘a’ there was one chamber on the top of the plot (subsequently referred to as area “a” top chamber); in the no-buffer control plots, there was an additional chamber near the bottom of the plot (called area “a” bottom chamber); (ii) in area “b” there were two chambers, one on the top and one on the bottom of the buffer strip (subsequently referred to as area “b” top and bottom chambers, respectively). Gas sampling was conducted periodically from May to October 2019, between 10:00 and 13:00, using 60-mL syringes and pre-evacuated 22-ml vials fitted with butyl rubber septa. At each sampling occasion, samples were collected at four-time intervals (0, 20, 40, and 60 min) from three chambers to account for the non-linear increase in gas concentration with deployment time (Grandy et al. 2006; Kaiser et al. 1996). The remaining chambers were sampled terminally at 40 min after closure (Chadwick et al. 2014). Additionally, ten ambient gas samples were collected adjacent to the experimental area: five at the start and another five at the end of each sampling event. CO_2_ concentrations were measured using a Perkin Elmer Clarus 500 gas chromatograph (Perkin Elmer Instruments, Beaconsfield, UK) fitted with an electron capture detector (ECD) after applying a 5-standard calibration.

##### CO_2_ flux determination and calculations

As suggested by Conen and Smith (2000), soil CO_2_ fluxes were calculated based on the rate of change in concentration (ppm) within the chamber, which was estimated as the slope of a linear regression between concentration and chamber closure time. Daily CO_2_ fluxes were computed using the Livingston and Hutchinson (1995) model. Cumulative CO_2_ fluxes were estimated by calculating the area under the gas flux curve after linear interpolation between sampling points (Mosier et al. 1996).

#### Soil analyses and meteorological variables

Soil pH [within-lab precision (RSD): 0.015] was measured using water (1:2.5) (Jenway pH meter, Staffordshire, UK), and soil organic matter (OM) was determined using the loss-on-ignition (LOI) technique (Wilke 2005). Composite soil samples (0–10 cm), made up of four random sub-samples, were collected monthly within 1-m of each chamber using a soil corer with a semi-cylindrical gouge auger (2–3 cm diameter) (Poulton et al. 2018). Total oxidized N [comprised of nitrite (NO_2_^−^) and nitrate (NO_3_^−^) N, the former considered to be negligible] and ammonium N (NH_4_^+^) [within-lab precision (RSD%): 7.2%] were quantified by extracting field-moist 20 g soil samples using 2 M KCl; 1:5 soil: extractant ratio, and analysis performed using an Aquakem™ analyzer (Thermo Fisher Scientific, Finland). At every gas-sampling occasion, composite soil samples (0–10 cm) made of four random sub-samples were collected within 1-m from each chamber using a soil corer for gravimetric soil moisture determination. Dry bulk density (BD) was determined at the start of the experiment next to each chamber using the core-cutter method (Amirinejad et al. 2011) and used to convert the gravimetric moisture determined during each of the gas sampling events into percent soil water-filled pore spaces (WFPS). Average daily precipitation was calculated from data measured at hourly intervals by an automatic weather station courtesy of the Environment Change Network (ECN) at Rowden, North Wyke (Lane 1997; Rennie et al. 2020).

### Data processing and statistical analysis

Linear mixed models in Genstat 20 (VSN International, Hemel Hempstead, United Kingdom) were used to determine whether cumulative CO_2_ differed with treatment. The random structure of each model (accounting for the experiment structure) was *block/plot/chamber*. The fixed structure (accounting for treatment effects) was treatment type/(treatment*distance). This model gives the following four tests in the output: (i) *Treatment type*—tests main maize cropped area vs. no-buffer control vs. riparian buffers, (ii) *Treatment type. treatment*—tests for differences between grass, willow, and woodland riparian buffers, (iii) *Treatment type. buffer distance*—tests for the difference between upper and lower riparian buffer areas, and (iv) *Treatment type. treatment. buffer distance*—tests for interaction between riparian buffer type and distance.

Linear mixed models with the same random and fixed structures as those used for CO_2_ were used to determine whether any measured soil variables (BD, pH, NH_4_^+^, TON, WFPS, and OM) differed with treatment. Pearson’s correlation coefficient (r) was used to indicate the strength of relationships between soil and environmental factors and CO_2_ emissions. This was tested more formally in the linear mixed models described above. If linear mixed models indicated that treatment differences were present, least significant differences (LSD) were calculated to determine which specific treatment pairs resulted in the significant differences in CO_2_ emissions. All graphs were generated using Sigma Plot (Systat Software Inc., CA, USA).

## Results

### Meteorological and soil characteristics

#### Rainfall patterns

The total rainfall for the whole experimental period was 492.2 mm, and the highest rainfall event of 118.2 mm received in October 2019. Before the highest rainfall in October, the second-highest rainfall events of 96.6 and 96.2 mm were recorded in June and September 2019, respectively.

#### Soil variables

Table 2 presents the average soil data during the experimental period. Soil pH ranged from 5.1 ± 0.17 and 5.5 ± 0.17, with the highest pH of 5.5 ± 0.17 (willow riparian buffer), which was however, not significantly (*LSD = 0.29*) different to the grass or woodland riparian buffers. The largest soil BD of 1.2 ± 0.05 g cm^− 3^ was recorded in the no-buffer control, which was not significantly different from the upslope maize and the different vegetated riparian buffers (*LSD = 0.19*). Soil OM ranged from 9.0 ± 3.2 to 17.8 ± 2.3%, with the largest %OM of 17.8 ± 2.3% recorded in the willow riparian buffer, which was, however, not significantly (*LSD = 8.6*) different to the woodland riparian buffer (15.98 ± 2.3%).

**Table 2:** Summary of soil parameters (mean ± standard error) in the upslope maize and downslope riparian buffers with different vegetation (upslope maize: *n* = 12, no-buffer control: *n* = 3 and each riparian buffer: *n* = 6) before the commencement of the current experiments in May 2019

Parameter	Upslope maize	No-buffer control	Grass Buffer	Willow buffer	Woodland Buffer	LSD
Soil pH	5.1 ± 0.17	5.1 ± 0.19	5.4 ± 0.17	5.5 ± 0.17	5.4 ± 0.17	0.29
Bulk density (g cm^− 3^)	1.21 ± 0.03	1.21 ± 0.05	1.1 ± 0.04	1.2 ± 0.04	1.2 ± 0.04	0.19
Organic matter (% w/w)	9.9 ± 1.3	9.0 ± 3.2	12.2 ± 2.3	17.8 ± 2.3	16.0 ± 2.3	8.6
NH_4_^+^-N (mg kg^− 1^ dry soil)	27.4 ± 2.98	20.6 ± 4.6	6.4 ± 2.7	13.6 ± 2.7	9.1 ± 2.7	7.8
TON (mg kg^− 1^ dry soil)	55.7 ± 1.7	42.8 ± 3.7	13.6 ± 3.0	4.99 ± 3.0	10.9 ± 3.0	10.0
WFPS (%)	86.9 ± 5.3	81.7 ± 9.9	86.7 ± 7.2	102.9 ± 7.2	98.2 ± 7.2	18.6

#### Soil mineral N-dynamics

**Fig. 1:**
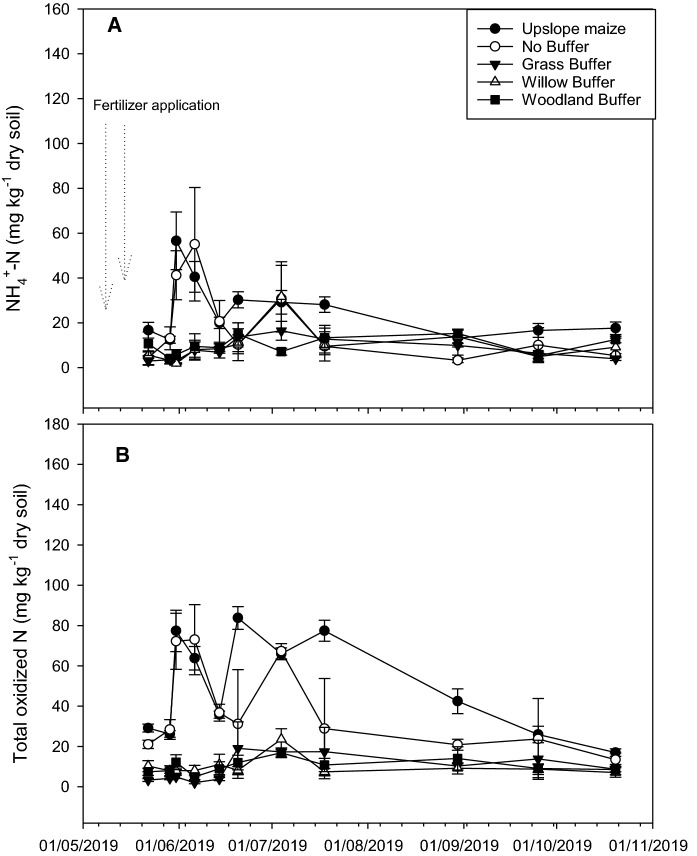
Soil NH_4_^+^ and TON in the upslope maize and downslope riparian buffers during the experimental period

Figure 1 shows soil mineral N dynamics during the experimental period. At the commencement of the experiment, NH_4_^+^-N was < 17 mg kg^− 1^ dry soil in all of the treatments, with the largest of 16.7 ± 3.5 mg kg^− 1^ dry soil observed in the upslope maize. However, after the second sampling event; which had been preceded by two fertilizer application events (Table 1), NH_4_^+^-N increased by almost 3-fold in the no-buffer control and upslope maize but remained relatively low in the vegetated riparian buffers. Despite the high NH_4_^+^-N values in the no-buffer control and upslope maize crop areas after fertilization, values dropped to < 30 mg kg^− 1^ dry soil after the fourth sampling event and remained low until the end of the experimental period. The average NH_4_^+^-N for the whole experimental period ranged from 6.4 ± 2.78 to 27.4 ± 2.8 mg kg^− 1^ dry soil, with the largest value of 27.4 ± 2.8 mg kg^− 1^ dry soil obtained from the upslope maize, which was however, not significantly (*LSD = 7.8*) different to the no-buffer control. It was, however, significantly different (*LSD = 7.8*) to the vegetated riparian buffers (Table 2).

Total oxidized N was < 30 mg kg^− 1^ dry soil in all of the treatments at the commencement of the experiment (Fig. 1). However, after the second sampling event, TON increased 4-fold in the upslope maize and no-buffer control but remained low in the riparian buffers. Despite a drop to ~ 35 mg kg^− 1^ dry soil in the upslope maize and no-buffer control during the fifth sampling event, the upslope maize emerged with the highest TON of ~ 81 mg kg^− 1^ dry soil during the sixth sampling event. However, these values dropped gradually up until the end of the experiment. Average TON for the whole experimental period ranged from 4.99 ± 3.0 to 55.7 ± 1.7 mg kg^− 1^ dry soil, with the highest value of 55.7 ± 1.7 mg kg^− 1^ dry soil obtained from the upslope maize. This was significantly different (*LSD = 10.0*) to all other treatments, except for the no-buffer control (Table 2).

#### %WFPS

**Fig. 2:**
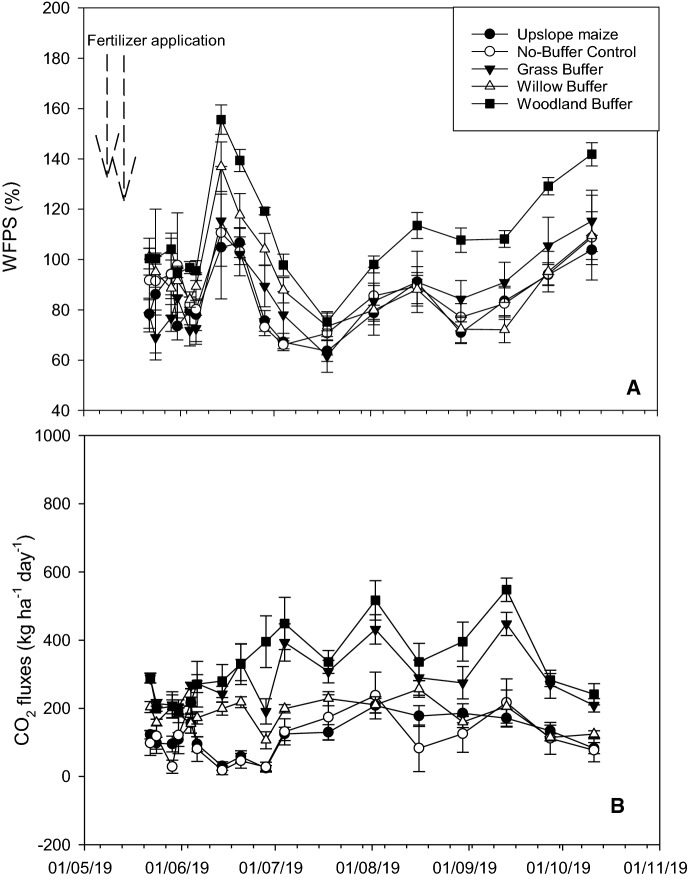
Daily **a** soil WFPS, and **b** soil CO_2_ fluxes, in the upslope maize and downslope riparian buffers. Data points and error bars represent the treatment means (cropland: *n* = 12, no-buffer control: *n* = 3, grass, woodland and willow buffer: *n* = 6) and SE during each sampling day

Soil WFPS trends during the experimental period are shown in Fig. 2a, and Table 2 shows the average %WFPS for the whole season. The highest %WFPS was observed during the fifth sampling event, with the overall highest estimate observed in the woodland riparian buffer treatment. The woodland riparian buffer maintained higher %WFPS values than the remainder of the treatments during the experiment. The average %WFPS for the whole experimental period ranged from 81.7 ± 9.9 to 102.9 ± 7.2%, with the highest value recorded in the willow riparian buffer, which was however not significantly (*LSD = 18.6*) different to the woodland riparian buffer treatment, or any of the other treatments.

### Treatment effects on explanatory variables

Table 3 shows that soil OM differed between sampling areas; upslope and downslope chambers (*P < 0.05*), but there was no evidence of any other treatment differences. Soil OM in the vegetated riparian buffers was different from the upslope maize but not to the no-buffer control, which was not different from the upslope maize. Soil NH_4_^+^-N also differed between areas, but there was no evidence of any other differences between treatments. The NH_4_^+^-N in the vegetated riparian buffer strips was different from the upslope maize and no-buffer control, whilst, the upslope maize and no-buffer control were not different from each other. Soil pH was different between areas, and there was also an interaction between treatments and the upper and lower buffer areas. The soil pH in the vegetated riparian buffer strips was different from the upslope maize and no-buffer control; but they were not different to each other. Soil pH was different in the upper and lower areas of the willow and woodland riparian buffer strips but not in the grass riparian buffer strips. TON was different between areas, but there was no evidence of any other treatment differences. All three riparian buffer vegetation types were different, and there was no evidence of any treatment differences for BD or WFPS (Table 2).

**Table 3:** P-values for tests from LMMs on each of the measured soil variables

Factors and interactions	OM	BD	NH_4_-N	pH	TON	WFPS
Area	0.04	0.29	< 0.001	< 0.001	< 0.001	0.23
Area * Treatment crop	0.31	0.13	0.16	0.238	0.173	0.24
Area * Buffer area	0.551	1	0.97	0.959	0.349	0.9
Area * Treatment crop * Buffer area	0.079	1	0.77	0.05	0.5	0.84

### CO_2_

#### CO_2_ fluxes

Figure 2b shows daily CO_2_ fluxes for the different treatment during the experimental period. CO_2_ fluxes were < 289.3 kg ha^− 1^ day^− 1^ at the commencement of the experiment, with the largest of 289.3 ± 14.5 kg ha^− 1^ day^− 1^ recorded in the woodland riparian buffer treatment. The woodland and grass riparian buffers maintained predominately larger (up to 547.9 ± 33.9 kg ha^− 1^ day^− 1^ from the woodland buffer on the 13th September 2019) whilst the willow riparian buffer, no-buffer control and upslope maize maintained lower fluxes throughout the experimental period. Prior to the larger peak, two smaller peaks of 449 ± 76.6 and 516.9 ± 57.9 kg ha^− 1^ day^− 1^ were observed on the woodland riparian buffer on the 4th of July and the 2nd of August 2019, respectively.

#### Cumulative CO_2_ emissions

**Fig. 3:**
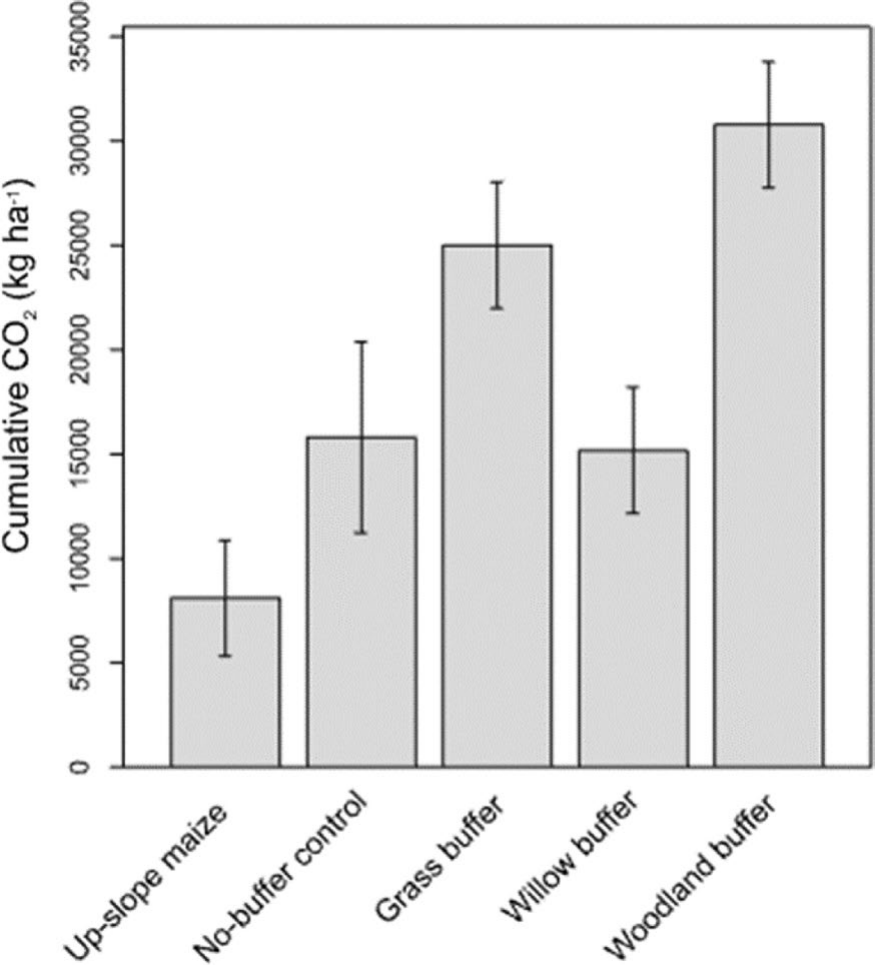
Cumulative CO_2_ emissions for the whole experimental period from the upslope maize and different downslope buffer vegetation. Error bars represent 95% confidence intervals (cropland: *n* = 12, no-buffer control: *n* = 3, grass, woodland and willow buffer: *n* = 6). Vertical lines are 95% confidence intervals

Figure 3 shows cumulative CO_2_ emissions in the descending order: woodland riparian buffer: 322.9 ± 3.1 kg ha^− 1^ > grass riparian buffer: 285 ± 2.7 kg ha^− 1^ > 182 ± 1.9 kg ha^− 1^ > upslope maize: 118 ± 2.0 kg ha^− 1^ > no buffer control: 112.7 ± 3.6 kg ha^− 1^. Significantly large (*p = < 0.0001*) emissions were obtained from the woodland riparian and grass riparian buffer treatments (not significant to each other) compared to the remainder of the treatments.

#### Relationships between cumulative CO_2_ emissions and measured soil variables

**Fig. 4:**
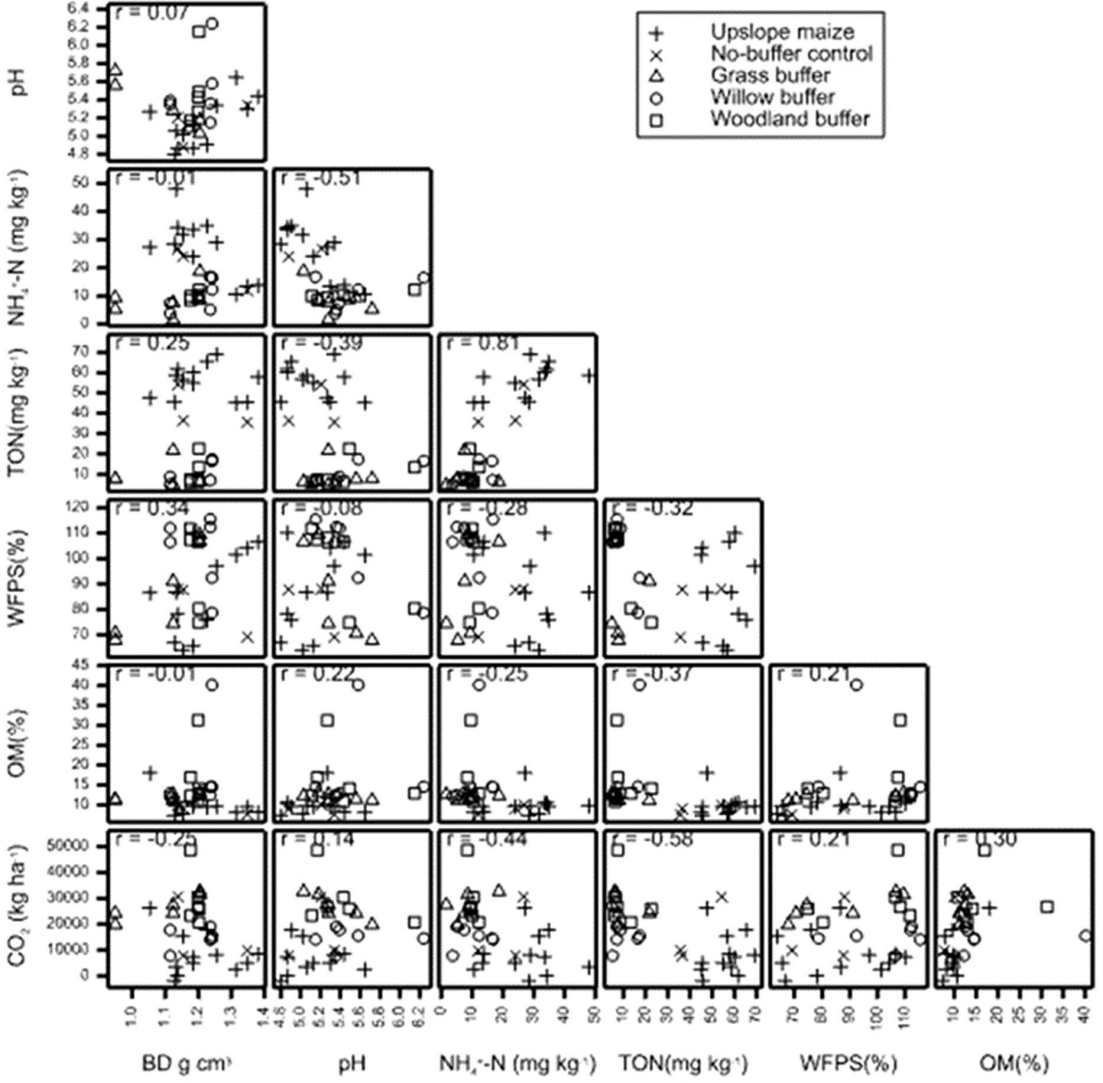
Scatterplot showing the relationships between the variables pH, soil NH_4_^+^-N, soil TON, water filled pore space (WFPS%), organic matter (OM), bulk density (BD) and cumulative CO_2_ emissions for the upslope maize and the downslope riparian buffers with different vegetation treatments. r = Pearson’s correlation coefficient

Soil pH (r = 0.14; *p = 0.03*), NH_4_^+^–N (r =− 0.44; *p = 0.003*), and TON (r = − 0.58; *p = < 0.0001*) have significant relationships with cumulative CO_2_ (Table 4; Fig. 4). Soil CO_2_ emissions showed to increase with increasing soil pH, OM, and %WFPS, and decreased with every increase in soil BD, NH_4_^+^–N, and TON.

**Table 4:** *P*-values for the slope of the fitted line of the model for CO_2_ and measured soil variables

Variable	Intercept	Standard error intercept	Slope	Standard error slope	*P-value*
BD	51,694	24,844	− 29,098	20934.2	0.177
pH	− 74,174	40,215	17,262	7531.9	0.030
NH_4_	26,065	4046	− 513.5	158.81	0.003
TON	25,805	2916	− 289.4	71.08	< 0.001
WFPS	− 417.9	10,318	194.4	111.72	0.098
OM	10,385	4328	543.2	287.85	0.071

## Discussion

### Soil CO_2_ emissions

#### Soil CO_2_ and environmental controls

Significantly higher CO_2_ were consistently measured in the grass and woodland riparian buffers similar to previous studies which compared soil respiration between croplands and riparian buffer systems particularly Tufekcioglu et al. (2001), and Jacinthe et al. (2015). The previous authors primarily linked the high CO_2_ fluxes in the vegetated riparian buffers compared to croplands to soil moisture and temperature differences as influenced by land-use differences of the two systems. In the current study, the consistently high CO_2_ fluxes in the woodland riparian buffer can be linked to the higher soil moisture it maintained throughout the experimental period (Fig. 2 A and B). Our findings consistent with other authors particularly Singh and Gupta (1977), Davidson et al. (1998), and Reth et al. (2005) who observed that high soil moisture regulated soil CO_2_ diffusion, hence its pronounced influences on soil respiration. Also, Sainju et al. (2010) reported a peak of CO_2_ fluxes immediately after a rainfall event (> 10 mm), which further highlights the role of soil moisture in CO_2_ production. We also observed an increase in CO_2_ with every increase in soil moisture in the current study.

Soil temperature is an environmental factor controlling CO_2_-producing microbial reactions provided that other factors including soil moisture and C contents are not limiting. For instance, Li et al. (2013) observed that only 26–34% of the seasonal variations in soil CO_2_ fluxes could be explained by soil temperature in exponential equations, implying that there were other factors affecting soil CO_2_. Thus, in the current experiment, the upslope maize and no-buffer control had a row crop which was mostly bare and hence prone to higher temperatures compared to the permanently ground covered-riparian buffers, but the latter treatments had low soil OM and consequently low CO_2_ fluxes. This then highlight the interactive role of soil C addition, temperature and soil moisture in CO_2_ production, similar to other authors, particularly Davidson et al. (1998), Epron et al. (1999), and Šimek et al. (2004)

Denitrification is a process carried out by facultative anaerobes and free energy, nitrogen gas (N_2_), and CO_2_ are produced as result of electron transfer between nitrate (NO_3_^−^) and C (Hume et al. 2002; Tusneem 1970). The process is highly dependent on the supply of readily available C and accounts for about 37% of the CO_2_ from the soil respiration systems (Ingersoll and Baker 1998; Rastogi et al. 2002). Thus, the predominantly higher soil moisture in the woodland riparian buffer coupled with high OM compared to the remainder of the treatments during the experimental period (Fig. 2 A and B) could have promoted denitrification in the treatment which increased CO_2_ fluxes similar to Beauchamp et al. (1989) and Dlamini et al. (2020).

#### Soil CO_2_ emissions in upslope maize and downslope riparian buffer strips

High CO_2_ emissions from the riparian buffers compared to croplands are linked to differences in biomass, C inputs and density of plant roots in the two systems (Jacinthe et al. 2015; Tufekcioglu et al. 2001). The relatively high soil OM in in the woodland riparian buffer may have resulted to increased C-priming effect hence the high CO_2_ emissions in the treatment, similar to findings by Šimek et al. (2004). Despite having the largest amount of soil OM, the willow riparian buffer had low CO_2_ emissions, which could mean that the treatment had a low labile C fraction similar to other studies including Dlamini et al. (2020), but we did not quantify C fractions in the current study. The previous author reported that treatments with highly labile C (readily available for microbial reactions) result to high CO_2_ compared to those with less labile C. Soil respiration is an indicator of total soil biological activity, and therefore an indicator of overall soil quality (Tufekcioglu et al. 2001; Visser and Parkinson 1992), and vegetated riparian buffers have been reported to improve soil quality characteristics compared to croplands (Salehin et al. 2020; Seobi et al. 2005; Udawatta et al. 2009), thus the resultant higher soil CO_2_ emission the grass and woodland riparian buffers compared to the upslope maize and no-buffer control of the current experiment.

## Conclusions

Our replicated plot-scale facility experiment showed that when different riparian buffer vegetation are introduced for water quality purposes in fodder maize production, the woodland and grass riparian buffers may pose a CO_2_ threat. Accordingly, our results attest to the unintended effects of some riparian buffers vegetation in emitting CO_2_, particularly when primarily implemented for water quality protection measures. The type of work undertaken in our experiment herein demonstrates the importance of gathering data for co-benefits and trade-offs associated with the management of agroecosystems.
